# Lessons Learned From Failures and Success Stories of HIV Breakthroughs: Are We Getting Closer to an HIV Cure?

**DOI:** 10.3389/fmicb.2020.00046

**Published:** 2020-01-31

**Authors:** V. Kalidasan, Kumitaa Theva Das

**Affiliations:** Infectomics Cluster, Advanced Medical and Dental Institute, Universiti Sains Malaysia, Kepala Batas, Malaysia

**Keywords:** HIV eradication and cure, Berlin patient, London patient, Düsseldorf patient, Boston patients, Essen patient, Mississippi baby, elite controllers

## Abstract

There is a continuous search for an HIV cure as the success of ART in blocking HIV replication and the role of CD4^+^ T cells in HIV pathogenesis and immunity do not entirely eradicate HIV. The Berlin patient, who is virus-free, serves as the best model for a ‘sterilizing cure’ and many experts are trying to mimic this approach in other patients. Although failures were reported among Boston and Essen patients, the setbacks have provided valuable lessons to strengthen cure strategies. Following the Berlin patient, two more patients known as London and Düsseldorf patients might be the second and third person to be cured of HIV. In all the cases, the patients underwent chemotherapy regimen due to malignancy and hematopoietic stem cell transplantation (HSCT) which required matching donors for CCR5Δ32 mutation – an approach that may not always be feasible. The emergence of newer technologies, such as long-acting slow-effective release ART (LASER ART) and CRISPR/Cas9 could potentially overcome the barriers due to HIV latency and persistency and eliminate the need for CCR5Δ32 mutation donor. Appreciating the failure and success stories learned from these HIV breakthroughs would provide some insight for future HIV eradication and cure strategies.

## Introduction

As of 2018, the Joint United Nations Programme on HIV/AIDS (UNAIDS) reported that 37.9 million people were living with HIV, of which 14.6 million people had no access to antiretroviral therapy (ART). This phenomenon is probably attributed to constrained health budgets and investments to effectively treat the HIV epidemic. About $301 billion was required to scale-up the therapy from 1995 to 2015 (Forsythe et al., 2019). A significant increase in investment is needed to enhance universal access to therapy and reduce the number of new infections in low- and middle-income countries (Deeks and Barré-Sinoussi, 2012). Based on that projection, $880 billion is needed for therapy to achieve the ‘90-90-90’ target by 2030.

This life-saving ART has public health benefits such as (Jain and Deeks, 2010): (i) preventing non-AIDS related morbidity and mortality, (ii) avoiding irreversible harm to the human immune system, and (iii) restricting the transmission of HIV to other individuals. Moreover, with early ART initiation in primary HIV infections, there would be slow disease progression and reduction in the size of the HIV reservoir (Chen et al., 2018). Early ART partially promotes restoration of HIV-associated gut-associated lymphoid tissues (GALT) damage and preserves the function of immune cells in both blood and gut. Despite the remarkable potency of ART, several issues persist, including (Bailey and Fisher, 2008; Desai et al., 2012; Solomon and Sax, 2015): (i) failure to eradicate latent reservoir cells, (ii) potential toxicities, short- and long-term side effects, (iii) negative drug-drug interactions, (iv) dependency on daily and strict adherence, (v) drug resistance and limited treatment options for multiclass resistance, (vi) limited availability of drug in resource-poor countries, and (vii) cost-ineffective and non-curative.

As a result, there is a constant pursuit for novel drugs and new approaches to eradicate the virus. Additional strategies have been implemented to prevent infection (Demberg and Robert-Guroff, 2012), such as: (i) syringe and needle exchange programs among intravenous drug users (IDU), (ii) oral/systematic pre-exposure prophylaxis (PrEP), (iii) post-exposure prophylaxis (PEP), (iv) gel base microbicides, (v) male circumcision, (vi) CCR5 co-receptor blocking molecules or permanently knocking out expression of CCR5, and (vii) “shock-and-kill” therapy using latency-reversing drugs such as HDAC inhibitors, to reduce the latent reservoirs in patients. Nevertheless, most of these interventions have their drawbacks, including cost-ineffectiveness, drug adherence and resistance, as well as side-effects/toxicities.

Thus, this review aims to unravel some of the critical aspects of an HIV cure by deciphering the failure and success stories of HIV breakthroughs. The lessons learned from each of the stories are also discussed extensively to understand the shortcomings and to discover clues for HIV eradication and cure.

## Hiv Eradication and Cure Research: Barriers and Approaches

**FIGURE 1:**
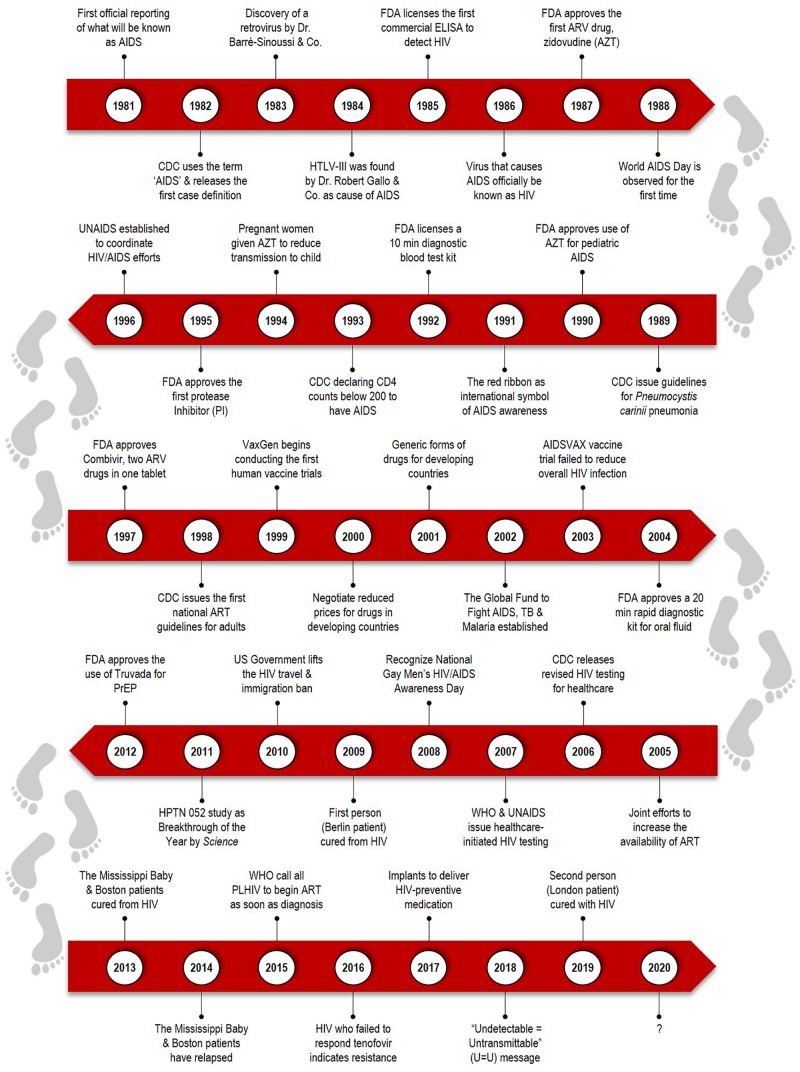
Timeline covers the highlights over the past three decades of HIV/AIDS. The history of HIV/AIDS epidemic begins from the first reported cases in 1981 of an unknown virus. The scientific and medical advances, such as the development of antiretroviral therapy (ART), cure breakthrough in Berlin patient, and other emerging initiatives for HIV eradication and cure strategies.

Revisiting the history of HIV/AIDS is necessary to fully appreciate the developments undertaken for HIV cure research (Figure 1). On 5 June 1981, the first official reporting of what is now known as AIDS was made, prompting the medical and scientific community to investigate the cause of this mysterious disease (Barré-Sinoussi et al., 2013). On 20 May 1983, a French virologist, Luc Montagnier identified a retrovirus called lymphadenopathy-associated virus (LAV) as the causative agent of AIDS (Schmid, 2018). At the same time, Robert Gallo announced the human T-cell lymphotropic viruses (HTLVs) as the cause of AIDS in April 1984, which was later found to be similar to Montagnier’s LAV. Finally, in 1986, the AIDS virus was officially named as the human immunodeficiency virus (HIV).

With the advancement of modern medicine, there are observable changes in the disease trend over the decades (Deeks et al., 2013). There used to be an exponential increase in new infections among young adults and children. Life expectancy was significantly reduced (less than 2 years) due to initiation of AIDS-defining illness, such as tuberculosis. Such comorbidity was due to the lack of access to ART and severe immune deficiency in untreated patients. In comparison, presently, more people are living with HIV, and it is more common among middle-aged individuals and lesser in children. More patients have access to ART, and life expectancy has increased. However, ART cannot fully restore immune deficiency, and there is an increase in HIV-related complications of cardiovascular, liver, renal, and cognitive function.

In 2012, the International AIDS Society (IAS) outlined seven key scientific priorities for developing a cure for HIV/AIDS in the year 2012 (Corbyn, 2012; Deeks et al., 2012) and were expanded to cater emerging issues. The new ‘Global Scientific Strategy: Toward an HIV Cure’ that was introduced in 2016 (Deeks et al., 2016) described: (i) molecular biology of HIV latency, (ii) immunology of HIV persistence, (iii) models for HIV cure or sustainable remission, (iv) remission in the pediatric population, (v) gene and cell therapy, (vi) novel biomarkers to quantify HIV persistence, and (vii) social science and health systems research. A trinity of HIV eradication and cure strategies highlighted the importance of Zhang and Crumpacker (2013): (i) defining CD4^+^ T cells in HIV pathogenesis and immunity, (ii) development of ART to block HIV replication, and (iii) studying the ancient human endogenous retroviruses (HERVs) in human genome. This model aimed to regulate memory CD4^+^ T cells turnover and eliminate HIV latency, the transcriptionally silent integrated HIV DNA in long-lived CD4^+^ T cells. Reprograming the memory CD4^+^ T cells repertoire and improving host antiviral immunity could tackle barrier related to HIV latency.

Chronic immune activation is the hallmark feature of HIV infection, marked by activation markers on lymphocytes, polyclonal expansion of B cells, elevated T cell turnover, and increased proinflammatory cytokines and chemokines in serum (Khaitan and Unutmaz, 2011). Even though immune cells are activated, they fail to respond against HIV, probably due to immune exhaustion. Immune exhaustion occurs as a result of persistent antigenic stimulation during infection, resulting in a loss of proliferative capacity of memory CD4^+^ T cells. This continuous antigenic stimulation leads to hyperimmune activation, characterized by upregulated expression of inhibitory receptors such as the cytotoxic T-lymphocyte-associated protein 4 (CTLA-4), programed cell death-1 (PD-1), T-cell immunoglobulin mucin 3 (TIM3), and lymphocyte-activation gene 3 (LAG-3) (El-Far et al., 2008; Kahan et al., 2015). Exhausted CD4^+^ T cells in return cause CD8^+^ T dysfunction, thereby leading to decreased viral control and continued disease progression.

Another major constraint for HIV eradication and cure research is the HIV persistence in ART suppressed individuals. Even though a steady-state level of a very-low viremia was reported among those on ART, the long-term persistence occurs over time, and is not only due to reactivation of the latent virus in specific cells (Martinez-Picado and Deeks, 2016; Peluso et al., 2019). There are a few plausible reasons for the underlying persistency (Kulpa and Chomont, 2015; Mzingwane and Tiemessen, 2017): (i) residual levels of replicative-competent virus that may not be entirely suppressed in drug-penetrable anatomical compartments, (ii) presence of a small pool of cells carrying silent integrated genomes that can potentially reactivate (latent virus), (iii) continuous immune exhaustion and inflammation that fail at controlling virus replication in infected cells, and (iv) cell-to-cell transmission in tissues that protects the virus from the effect of ART.

Currently, two approaches can be undertaken for an HIV cure. Firstly, a ‘sterilizing cure’ eliminates all HIV-infected cells, and secondly, a ‘functional cure’ is long-term control of HIV in the absence of ART (Lewin et al., 2011). These two cure strategies differ in terms of its treatment model and the HIV RNA level that can be achieved. For sterilizing cure (also known as infectious disease model), all HIV-infected cells are treated and eliminated so that the individual has HIV RNA equivalent to or less than one copy/ml. An example of this approach would be the Berlin patient who appeared to be free of his malignancy and HIV infection. On the other hand, functional cure (also known as cancer model), aims for remission in the absence of treatment, with HIV RNA less than 50 copies/ml. Elite controllers are the only group of people where HIV RNA remains below the detection level for an extended period without ART.

At this juncture, this brings up the question of what type of cure can ultimately be achieved. Sterilizing cure has its drawbacks, such as finding matched donors and potential transplantation complications (Xu et al., 2017). Thus, most of the scientific community believe that a sterilizing cure would not be feasible, and a functional cure is more achievable (Sylla et al., 2018).

## Hiv Cure Failures: Lessons Learned

Looking back to 1984, the US Secretary of Health and Human Services, Margaret Heckler, declared a vaccine would be found within 2 years of the discovery of HIV/AIDS. In reality, treatment is far more challenging than what was first thought, even though many strategies such as vaccine trials and HSCT have been executed. Here, we discuss some of the pitfalls throughout the HIV cure journey, and the lesson learned from these setbacks.

### AIDSVAX^®^ Vaccine Trials

In the 1990s, VaxGen (San Francisco, CA, United States) developed AIDSVAX^®^, the world’s first Phase III clinical trial of an HIV/AIDS vaccine. The trials were double-blinded and placebo-controlled, where the vaccine arm volunteers received AIDSVAX^®^, while the placebo arm volunteers received alum (Adis Editorial, 2003). The study volunteers were vaccinated seven times (0, 1, 6, 12, 18, 24, and 30 months), with intramuscular 300 μg injections, and the booster doses aimed to stimulate high antibody levels. The volunteers also received risk-reduction counseling, were asked a series of clinical questions, and had their blood drawn for serological testing.

The vaccine trials took place in two sites (Johnston, 2003). VAX004 study was conducted in North America and the Netherlands. The study was started in June 1998 and was fully enrolled in October 1999, with 5,400 volunteers comprising of 5,100 men who have sex with men (MSM) and 300 women with multiple sex partners. In a 2:1 ratio, volunteers received either AIDSVAX^®^ B/B vaccine (rgp120/HIV-1, subtype B clade virus) or placebo (alum). On the other hand, VAX003 study in Thailand was started in March 1999 and fully enrolled in August 2000, with 2,500 volunteers comprising of men and women who were intravenous drug users (IVDUs). In a 1:1 ratio, volunteers received either AIDSVAX^®^ B/E vaccine (rgp120/HIV-1, subtype B and E clade viruses) or placebo (alum).

The outcome of the vaccine trials was disappointing (Ready, 2003). Even though the vaccine was well-tolerated, the VAX004 study did not show any HIV-1 prevention (The rgp 120 H I V Vaccine Study Group, 2005). High infection rates (6.7% in vaccine arm) in all racial subgroups were correlated with lower vaccine-induced antibody response, with overall vaccine efficacy (VE) estimate of 6% (Gilbert et al., 2005). Similarly, the VAX003 trial also neither prevented HIV-1 infection nor delayed HIV-1 disease progression, as there were no significant differences between the two treatment arms (McCarthy, 2003; Pitisuttithum et al., 2006). VE was estimated at 0.1%, which suggested that the vaccine was not effective against either subtype B or E clade viruses.

Other than VAX004 (phase III), and VAX003 (phase III), four other HIV-1 vaccine trials include HVTN 502 step trial (phase IIb), RV144 (phase III), HVTN 503 Phambili trial (phase IIb) and HVTN 505 (phase IIb) (Ensoli et al., 2014; Excler and Michael, 2016). Compared to the other trials, only RV144 trial [ALVAC-HIV (vCP1521) and booster AIDSVAX^®^ B/E] vaccine showed modest VE (31.2%), but the vaccine did not affect the viral load or CD4^+^ in HIV-1 diagnosed patients (Rerks-Ngarm et al., 2009). Although the AIDSVAX^®^ failed to protect HIV-1 infected individuals, from these trials, we can appreciate the initiatives required to execute an effective vaccine plan. AIDSVAX^®^ trials proved that it is possible to recruit, counsel, immunize and closely follow-up with participants for more than 30 months with established collaborations, network, staff and facilities (Pitisuttithum, 2008).

Lessons learned from these trials, and new priorities that should be considered in HIV-1 vaccine development are summarized as follows (McMichael and Haynes, 2012; Su and Moog, 2014; Excler and Michael, 2016; Pollara et al., 2017): (i) induction of broadly neutralizing antibody (bNAbs) could neutralize multiple HIV-1 isolates, (ii) great neutralizing breadth and immunogenetic characteristic of bNAbs, (iii) stimulated expansion of B cells favor development of bNAbs precursors, (iv) elicitation of non-neutralizing Fc receptor (FcR)-mediated effector functions, in the absence of bNAbs production (v) increased ADCC activity in the presence of low circulating Env-specific IgA, (vi) increased antibody-dependent cell-mediated virus inhibition (ADCVI) activity via HIV-specific antibody, and (vii) stimulation of more effective CD8^+^ and CD4^+^ T cell responses. While the priorities are challenging to achieve in vaccines, there are alternative methods that can be applied directly to infected individuals.

### Mississippi Baby

An infant at 8 months of gestation was born to a woman with HIV-1 infection, who was not on ART or any other antenatal care during pregnancy, as the mother was found to be HIV-1 positive only during labor (Check Hayden, 2013b; Persaud et al., 2013). The mother’s initial viral load (24 h) was 2423 copies/ml, followed by 6763 copies/ml after 26 months of delivery. There were 137 infectious units per million of replication-competent virus in infected the woman’s CD4^+^ T cells. Both the mother and the infant did not have any protective HLA alleles and had wild-type CCR5 co-receptor. The following regimen of ART was administered to the infant: (i) zidovudine (AZT) at 30 h of age, (ii) AZT + lamivudine (3TC) + nevirapine (NVP) at 31 h of age, and (ii) AZT + 3TC + ritonavir-boosted lopinavir (LPV/r) at 11 days of age. The HIV-1 RNA level measurement during ART was: (i) 19,812 copies/ml at 31 h of age, (ii) 2617 copies/ml at 6 days of age, (iii) 516 copies/ml at 11 days of age, (iv) 265 copies/ml at 19 days of age, and (v) less than 48 copies/ml at 29 days of age. The child missed several clinical appointments between 18 and 23 months of age, and during 18 months of age, the child was not on ART. Interestingly, the plasma HIV-1 RNA was undetectable, and the PCR test was negative at 24 months of age. Furthermore, circulating HIV-1 antibodies were not detected and resting CD4^+^ T cells did not yield replication-competent viruses.

This news was undoubtedly a tremendous miracle and suggested that very early ART administration potentially interferes with the development of memory T cells and latent reservoir (Dolgin, 2013). Even though this action was deemed an aggressive tactic, it was crucial to determine if a similar treatment style could be employed in other HIV-infected babies globally. Shockingly, in July 2014, using an enzyme-linked immunosorbent assay (ELISA), HIV-1 specific antibody and cell-mediated immune response demonstrated viremic rebound and Mississippi Baby was reported as not ‘cured’ (Ledford, 2014; Luzuriaga et al., 2015). Moreover, Western blot analysis revealed reactivity to HIV-1 Env (gp160, gp120, and gp41), Gag (p55, p24, and p17) and Pol (p66). Amplification and sequencing of full-length HIV-1 Env showed 98.6% sequence similarity to maternal plasma viral sequence collected 24 months after delivery. This evidence proved that there was an occurrence of latency during viral quiescence in a small pool of resting memory CD4^+^ T cells in the toddler (Siliciano and Siliciano, 2014). Even though it was small, the stable pool of T cells could carry an integrated but transcriptional silent viral genome, which in turn could be replication-competent virus. Overall, this phenomenon provided insight that early ART may suppress HIV, but not completely eradicate the latent reservoirs (Ananworanich and Robb, 2014). Viremic rebound can occur spontaneously, and it is unpredictable after ART interruption. Most importantly, at that time, the assay to quantify latent reservoir was not sensitive enough to determine HIV persistency.

Other than the Mississippi baby, two clinical cases investigating the potential of early ART administration for perinatally HIV-1-infected newborns were reported. The Canadian and Milan babies were treated with ART within the first 24 and 12 h of their lives respectively and appeared to be stable without the presence of replication-competent viruses for almost 3 years. After ART interruption, the viral load increased drastically (Ananworanich, 2015). From these cases, the administration of ART as early as possible seemed to be a good strategy, but treatment without interruption is worth considering. An appropriate starting regimen is also challenging (Luzuriaga et al., 2004). Even though three ART regimens (AZT + 3TC + NVP) were somewhat safe, effective and well-tolerated, a higher dose was prescribed for treatment rather than prophylaxis (Shah et al., 2014). Furthermore, due to the presence of inactive ingredients propylene glycol and ethanol in LPV/r, there is a higher risk of adverse effects such as hyperosmolality (with or without lactic acidosis), renal toxicity, central nervous system (CNS) depression, seizures, hypotonia, cardiac arrhythmias, electrocardiogram (ECG) changes and hemolysis (Nuttall, 2015).

Overall, from these HIV-1 infected infants’ cases, some of the critical aspects are (Luzuriaga et al., 2000, 2014; Bitnun et al., 2014; Cotton and Rabie, 2015): (i) early diagnosis and initiation of ART and adequate follow-up would delay HIV disease progression, (ii) suppression of virus replication might limit the priming and expansion of virus-specific immune responses (i.e., reduced efficiency of antigen presentation, limited ability to produce cytokines, lack of maintenance of cell-mediated immunity), (iii) size of the HIV-1 reservoir at birth is likely to be extremely small, but a stable pool of memory CD4^+^ T cells may be present, (iv) limitation to recover adequate numbers of peripheral blood mononuclear cells (PBMCs) to reasonably exclude the presence of replication-competent virus, (v) low number of cell replicates tested resulting in undetectable level of HIV-1 DNA in the CD4^+^ T cells, and (vi) a structured treatment interruption should be planned, as it is not feasible to examine every cell in infected infant to determine if HIV eradication has taken place. There may also be more factors at play in achieving an HIV cure, which may only be obvious when studying the efforts in infected adult patients.

### Boston Patients

The impact of allogeneic HSCT on peripheral blood viral reservoir was examined in two male HIV-1 adults, and the protective effects of ART on donor cells were observed (Henrich et al., 2013). Patient A acquired HIV-1 perinatally and was diagnosed with nodular sclerosing Hodgkin lymphoma (stage IV) in the year 2006. He was on a chemotherapy regimen of adriamycin, bleomycin, vinblastine, dacarbazine (ABVD), but experienced relapse, and salvage chemotherapy of ifosfamide, carboplatin, etoposide (ICE) was initiated. Unfortunately, his lymphoma recurred despite autologous stem cell transplantation, and he was administered with another salvage chemotherapy (gemcitabine, navelbine, doxorubicin). After complete remission, a single (7 out of 8) HLA-C-mismatched, reduced-intensity conditioning (RIC) chemotherapy HSCT was given from an unrelated donor. He was on ART (tenofovir, emtricitabine, efavirenz) 3–4 years before transplantation and continued the same regimen during and after transplantation. Mini-methotrexate, sirolimus, and tacrolimus were administered to prevent graft-versus-host disease (GVHD). 100% of peripheral blood granulocytes (at day 143 after transplantation) and 100% of CD3^+^ T cells (at day 216 after transplantation) were donor-derived. Despite the regimen, the patient developed chronic GVHD with skin, eye, and liver involvement approximately after 9 months and was treated with prednisone. Due to sclerodermatous chronic GVHD, he was on extracorporeal photopheresis, with excellent response to the procedure.

The CD4^+^ T cells following HSCT were significantly reduced initially but appeared to return to levels similar to prior transplantation. Before transplantation, patient A had 144 copies of HIV-1 DNA per 10^6^ PBMCs, and it was reduced to 87 copies/10^6^ PBMCs after transplantation (day 64). Interestingly, no HIV-1 DNA or p24 antigen was detected on day 1266 after transplantation. Plasma HIV-1 RNA was not detected before transplantation (day 6), 65 copies/ml after transplantation (day 143), followed by undetectable after transplantation (day 1077). The level of HIV antibody decreased 5-fold after transplantation.

Patient B acquired HIV-1 through sexual transmission and was diagnosed with diffused large B-cell lymphoma in the year 2003. He was on a chemotherapy regimen (cyclophosphamide, doxorubicin, vincristine, prednisone with rituximab – R-CHOP), alongside with ART (efavirenz-based combination). Unluckily, he was diagnosed with new mixed cellularity Hodgkin disease (stage IV) and was on ABVD chemotherapy, followed by salvage ICE chemotherapy due to recurrence, and finally received autologous HSCT in the year 2007. He developed persistent thrombocytopenia and anemia and was diagnosed with myelodysplastic syndrome (multilineage dysplasia). He was given a matched, sibling-donor RIC allogeneic HSCT and sirolimus, tacrolimus, and mini-methotrexate were administered to prevent GVHD. The ART regimen was changed to tenofovir, emtricitabine, nelfinavir, and abacavir because of detectable viremia and drug resistance mutations, prior to RIC HSCT. The patient developed GVHD of the skin, liver, and oropharynx, which required intermittent prednisone therapy. 100% of all leukocytes (including lymphocytes) were donor-derived. Even though the patient’s viral load was undetectable, the ART was changed to tenofovir, emtricitabine, and raltegravir before and after HSCT, to reduce drug-drug interactions.

The CD4^+^ T cells following HSCT were reduced but appeared to plateau. Before transplantation, patient B had 96 copies of HIV-1 DNA per 10^6^ PBMCs, and it increased to 281 copies/10^6^ PBMCs after transplantation (day 92). Similar to patient A, no HIV-1 DNA or p24 antigen was detected on day 652 after transplantation. Plasma HIV-1 RNA was not detected before transplantation (day 5) and after transplantation (day 59). The level of HIV antibody decreased 10-fold after transplantation. Both patients were heterozygous for the CCR5Δ32 mutation. Eventually, the mutation was lost after the patients achieved full donor chimerism.

Allogenic HSCT during ART potentially reduces HIV-1 reservoir, due to full donor chimerism and recovery of CD4^+^ T cells. Furthermore, the achievement in Boston patients suggests that it is possible to significantly reduce HIV latency, without the need for a donor with homozygous CCR5Δ32 mutation as long as ART continues to protect donor cells from the virus (Cannon et al., 2014). In terms of the procedure, the patients did not receive total body radiation or on anti-thymocyte globulin, unlike other cases reported elsewhere. The GVHD response may also have cleared the latent or active viruses in the patients. Similar to the Mississippi baby, the Boston patients demonstrate the absence of HIV-1 RNA, proviral HIV-1 DNA, viral outgrowth in plasma and PBMCs after years of transplantation, as well as lacked HIV-specific immune responses (Rainwater-Lovett et al., 2015).

The crucial question then is: Would Boston patients also face viral rebound, similar to the Mississippi baby, if treatment was interrupted? The simple answer is yes. Both patients developed detectable virus in their blood and were present with acute retroviral syndrome upon ART interruption (Check Hayden, 2013a; Henrich et al., 2014). Viremia in patient A was first detected 84 days after ART interruption. Although he was asymptomatic, he was put back on his previous ART regimen. At day 117, after cessation, his viral load was 4.2 million copies/ml, he had developed a new efavirenz-resistant mutation and was resistance to some protease inhibitors. He was prescribed a new ART regimen (tenofovir + emtricitabine + raltegravir + ritonavir-boosted darunavir) to manage his condition. At day 120, he was found to have low-grade lymphocytic pleocytosis consistent with HIV-associated meningitis; however, HIV-1 RNA was not apparent in cerebrospinal fluid (CSF). He continued to face acute retroviral syndrome and some medication side-effects, and it was finally resolved with reduced HIV-1 RNA level similar to before ART initiation.

On the other hand, patient B presented with consistent HIV-1 negative results during ART interruption. Unfortunately, 225 days after cessation, he showed worsening symptoms and was found to have an HIV-1 RNA level of 1.9 million copies/ml. He was immediately put on his ART regimen and plasma viremia reduced, with no drug resistance. Unlike patient A, his CSF had HIV-1 RNA levels of 269 copies/ml. The near-full-length HIV-1 envelope sequence from the viral rebound in both patients was related to HIV-1 DNA sequences before HSCT. The treatment interruption gave rise to some appreciable lessons (Kuritzkes, 2016): (i) HSCT may have decreased HIV-1 reservoir, but there was some degree of virus persistency in infected cell or tissue, (ii) a small number of infected cells seemed to be sufficient to trigger HIV-1 replication upon ART interruption, (iii) limited sensitivity of assay to detect tissue reservoirs that persist in compartments of the cells, (iv) the longer the interval between HSCT and ART interruption, the longer the period the HIV remission would have taken place, and (v) newer approaches to assess the extent of HIV-1 reservoir depletion after therapeutic interventions should be considered.

### Essen Patient

A 27-year-old patient diagnosed with HIV-1 infection and anaplastic large-cell lymphoma was given HSCT from a donor who was homozygous for CCR5Δ32 mutation (Kordelas et al., 2014). The HIV-1 tropism was determined by genotyping the V3 amino acid sequence, and viral co-receptor usage was predicted using geno2pheno bioinformatic software. The HIV-1 tropisms profiles before stem cell transplantation were as follows: (i) 287 days prior to HSCT, HIV-1 tropism to CCR5 was found to be intermediate (false positive rate – FPR = 8.2%, RNA) before ART initiation (lopinavir-ritonavir, tenofovir, emtricitabine), (ii) 103 days prior to HSCT, R5-tropic HIV-1 tropism (FPR = 24.7%, RNA) and intermediate HIV-1 tropism (FPR = 6.6%, DNA), and (iii) 18 days prior to HSCT, X4-tropic HIV-1 tropism (FPR = 4.4%, DNA).

The patient’s ART was stopped before stem cell transplantation, and 20 days after HSCT, X4-tropic HIV-1 tropism (FPR = 0.4%) was observed. The previous ART regimen was resumed due to viremic rebound of 93,390 copies/ml of HIV-1 RNA in the patient after HSCT. In December 2012, the regimen was switched to lopinavir-ritonavir, lamivudine, and abacavir, and in April and May 2013, it was changed to lamivudine, abacavir, and raltegravir, resulting in HIV suppression. Once again, the ART regimen was interrupted due to relapse of T cell lymphoma. The case came to an end when the patient died with 7,582,496 copies/ml of HIV-1 RNA in the same year.

A deep sequence analysis of the HIV envelope gene from stored samples was done to understand the underlying mechanism of viral escape seen in the Essen patient (Verheyen et al., 2019). After HSCT, all HIV-1 RNA and proviral DNA sequences were found to be X4-tropic virus and genetically different from the R5-tropic virus. Most importantly, X4-tropic virus was already present in proviral DNA 103 days before HSCT. Driven by the loss of CCR5 co-receptor, there was a shift from the dominant CCR5-tropic virus before HSCT to CXCR4-tropic virus after transplantation. Thus, it is essential to note that CCR5 knockout strategies to control HIV-1 infection may not be the right approach.

The rapid rebound after HSCT by the highly replicative CXCR4-tropic virus in Essen patient has taught us some valuable lessons (Hütter, 2014; Hütter et al., 2015): (i) ART interruption prior to HSCT gave ample time to the virus to evolve to use alternative co-receptors, (ii) there is a higher probability of the V3 region of the virus to change from R5 to X4 co-receptor, (iii) a co-receptor switch is one of the viral escape mechanisms following HSCT of a homozygous CCR5Δ32 mutation, (iv) a complete replacement of patients CCR5-positive cells by CCR5Δ32 mutated donor cells did not occur, (v) the ART regimen should have been continued during HSCT, until donor chimerism is achieved. Thus, guidelines for ART regimen during chemotherapy and HSCT should be developed for better therapeutic outcomes.

## Hiv Cure Success: Lessons Learned

Despite the stories of HIV cure failures, the lessons learned are of utmost value to further strengthen HIV eradication. The Berlin patient is considered the only person to have been cured of HIV and has set a positive benchmark that led to subsequent studies hoping to replicate the results. Here, we discuss some of the achievements in HIV cure research, where these cases have shown the undetectable virus in the absence of ART.

### Berlin Patient

A 40-year-old white man, known as Timothy Ray Brown, lived with HIV-1 infection for about 10 years (Hütter et al., 2009; Brown, 2015). He was on ART regimen for 4 years (efavirenz, emtricitabine, tenofovir), with CD4^+^ T cell of 415/mm^3^ and undetectable HIV-1 RNA. He was diagnosed with acute myeloid leukaemia (AML) and was put on two courses of induction and one course of consolidation chemotherapy. Due to severe hepatic toxic effects, ART was interrupted, resulting in viral rebound (6.9 × 10^6^ copies/ml of HIV-1 RNA). However, ART was resumed immediately, and 3 months later, his viral load was undetectable again. Unfortunately, his acute myeloid leukemia relapsed, and he was given HSCT with CD34^+^ peripheral-blood stem cell from an HLA-identical (10/10) donor who had been screened for homozygous CCR5Δ32 mutation.

Brown received the graft, and anti-thymocyte globulin, cyclosporine, and mycophenolate mofetil to prevent GVHD, as well as on ART before the procedure. Although the engraftment was achieved 13 days after the procedure, his AML relapsed, and he underwent reinduction therapy (cytarabine, gemtuzumab). On day 391, he received HSCT again from the same donor, with a single dose of whole-body irradiation, which ultimately led to complete eradication of his AML. Two years after the transplantation, HIV-1 RNA and proviral DNA were no longer found in his blood, bone marrow or rectal mucosa using PCR assays (Kuritzkes, 2016; Martinez-Vesga, 2018). This phenomenon concluded that the donor-derived cells (CCR5Δ32 mutation) had successfully replaced Brown’s HIV-1 infected cells.

Even though he carried X4-tropic co-receptor (2.7–9.3% FPR), remarkably, the virus did not seem to rebound after HSCT in the absence of ART (Symons et al., 2014). Compared to Essen patient, there was a contradictory response to viral tropic shift. Although Essen patient exhibited a very minimal 0.4% FPR HIV-1 tropism to CXCR4 co-receptor (FPR cutoff in Berlin patient is 2.7%), that did not seem to protect the Essen patient. This phenomenon could be contributed to the dependency of the virus on CCR5 for multiplication, and high genetic barrier that conferred resistance toward CXCR4 co-receptor. Moreover, there was lesser number of residual CCR5-expressing CD4^+^ T cell after transplantation to support replication of CCR5-tropic variant in the Berlin patient. Furthermore, the Berlin patient received double transplantation whereas the Essen patient only once, so HIV eradication might have taken place even before the second transplantation (Hütter et al., 2015). Most importantly, Berlin patient was on ART interruption the day after his second transplantation, unlike Essen patient, whom ART was interrupted a week before transplantation, suggesting that ART continuation is crucial even during condition therapy until stable engraftment is confirmed.

Despite intense conflicts and challenges, the Berlin patient remains without any evidence of virus for about 10 years. Valuable lessons learned from this success story (Allers et al., 2011; Mitsuyasu, 2013; Yukl et al., 2013; Peterson and Kiem, 2019) include: (i) GVHD in which donor-derived immune response subsequently clears persistent HIV-1 infected cells, resulting in reduced HIV reservoir, (ii) application of anti-cancer strategies, such as cytotoxic drugs (cyclophosphamide, busulfan), chemotherapy agents (anti-thymocyte globulin, anti-myeloid leukemia monoclonal antibody) to eliminate HIV-infected cells, and (iii) not all patients who have undergone CCR5Δ32 mutation HSCT will achieve a conceivable cure, such as the Essen patient, which suggests that personalized strategies are needed. The Berlin patient is the first person to be cured of HIV infection and is the best living example for HIV eradication and cure research.

### London Patient

A man has been living with HIV-1 since 2003, with CD4^+^ T cell of 290/mm^3^, viral load of 180,000 copies/ml and was homozygous for wild-type CCR5 (Gupta et al., 2019). His ART regimen (tenofovir disoproxil fumarate, emtricitabine, efavirenz) was initiated in the year 2012. He was diagnosed with nodular sclerosing Hodgkin lymphoma (stage IVb) at the end of 2012 and was put on a chemotherapy regimen (doxorubicin, bleomycin, vincristine, dacarbazine), and salvage chemotherapy (etoposide, methylprednisolone, cytarabine, cisplatin, brentuximab, mini-LEAM – lomustine, etoposide, cytarabine. melphalan). The ART regimen was changed to tenofovir disoproxil fumarate, emtricitabine, and raltegravir during periods of the chemotherapy. Five-days of ART interruption was reported during late 2015 in which the viral load did not rise to set point. Drug resistance happened resulting in the switching of ART (rilpivirine, lamivudine, dolutegravir), and subsequent viral suppression took place.

The patient was then subjected to HSCT. No fully matched donors were identified; however, one allelic mismatch at HLA-B, who carried CCR5Δ32 mutation was found. He received the graft and was on a conditioning regimen (LACE – lomustine, ara-C, cyclophosphamide, andetoposide). Cyclosporine-A and methotrexate were administered to prevent GVHD, and his previous ART regimen was continued throughout the procedure. The patient developed a fever and gastrointestinal GVHD at day 77 that was resolved without any intervention. At day 85 after transplantation, he was treated for reactivation of Epstein–Barr virus and cytomegalovirus (CMV). Full donor chimerism was achieved, he carried CCR5Δ32 mutation, his CD4^+^ and CD8^+^ T cells loss CCR5 co-receptor and he could not be infected with CCR5-tropic viruses.

His ART regimen was maintained after HSCT and interruption was started at day 510. The viral load, HIV-1 DNA, and HIV-1 RNA were undetectable, despite repeated testing. HIV reservoir before transplantation (day 217, with ART) showed less than 0.286 infectious units per million T cells, while after transplantation (day 876, without ART) it appeared to be less than 0.063 infectious units per million T cells. Sadly, CXCR4-tropic HIV-1 virus *in vitro* challenge resulted in an infection in the patient’s cells, indicating risk of infection and the need for continuous monitoring. Both antibody and T cell responses appeared similar to the Berlin patient, which might have been contributed by GVHD prophylaxis and response. The comparable difference includes the conditioning regimen, where London patient received reduced-intensity chemotherapy agents, while the Berlin patient received total body irradiation and cyclophosphamide (Peterson and Kiem, 2019). Moreover, Brown was treated with anti-thymocyte globulin, whereas London patient with the anti-CD52 antibody. Single HSCT versus double HSCT would be another point of difference.

Although another breakthrough was achieved, it is still premature to conclude an HIV cure, as it has only been a short duration since its announcement at the Conference on Retroviruses and Opportunistic Infections in Seattle, WN, United States, in March 2019 (Cohen, 2019a; Kirby, 2019). London patient has provided an example of a scalable approach (Kirby, 2019; Saez-Cirion and Müller-Trutwin, 2019; Scarborough et al., 2019): (i) single HSCT with CCR5Δ32 mutated donor cells without the need of total body irradiation is adequate, (ii) GVHD reaction and sustaining full donor chimerism could be a vital event related to HIV-1 clearing, and (iii) newly replenished host cells with CCR5Δ32 donor cells promote remission through a ‘kill-and-block’ strategy. Currently, CCR5 gene therapy strategies using stems cells could be a new priority for treatment development, but require an optimization and thorough investigation to cater a large number of patients.

### Düsseldorf Patient

A 49-year-old man diagnosed with AML (Jensen et al., 2019) had an HIV-1 viral load of 29,400 copies/ml, and all HIV-1 associated proteins in his body. His ART regimen (tenofovir disoproxil fumarate, emtricitabine, darunavir) was initiated in 2011 and resulted in a decreased viral load. He received two courses of ICE and three courses of high dose cytarabine (HidAC) chemotherapy within the same year, resulting in remission. During this period his ART regimen was switched by replacing darunavir with raltegravir, and no drug resistance was found. Unfortunately, in 2012, his AML relapsed, and he was administered with a high dose of cytosine arabinoside and mitoxantrone (HAM), HidAC together with FluTreo conditioning regimen. Finally, in 2013 he received unmodified HSCT from a fully matched (10 out of 10) graft from a female CCR5Δ32 mutation donor.

However, his AML relapsed again around June 2013 and he was put on eight courses of azacytidine (5-AzaC) and four courses of donor lymphocyte infusion (DLI), resulting in complete remission of his AML, upon chimerism after 3 months (September 2013). He developed GVHD (responses not reported) within 2 years. His ART regimen was changed (abacavir, lamivudine, dolutegravir), and he remained on ART with undetectable viral load. After HSCT, his PBMCs, CSF, rectum, ileum, bone marrow, and lymph node as well as the viral outgrowth assays (VOA) were all negative for HIV-1. His HIV antibody profile was slightly positive to gp160, while other HIV markers were undetected. These patterns appeared to be similar to the London patient after HSCT with CCR5Δ32 mutation.

Prior to HSCT, the HIV-1 co-receptor usage was R5-tropic, and after HSCT, CCR5-negative HIV-specific CTL was found, suggesting complete replacement of patients CCR5-positive cells with CCR5Δ32 mutated donor cells. To determine whether HSCT had caused viral remission, ART interruption was initiated in November 2018, and no rebound has been seen so far (Scarborough et al., 2019).

The coincidental announcement of both the London and Düsseldorf patients’ cases were purely by chance (Saez-Cirion and Müller-Trutwin, 2019; The Lancet HIV, 2019). These achievements can be contributed to the IciStem program that consists of a cohort of patients with the malignant hematological disease and received HSCT. The program identified 22,000 potential donors who were homozygous for CCR5Δ32 mutation. Among 45 patients registered, 39 patients have undergone transplantation (of which 26 patients are still living), London and Düsseldorf patients were the only examples of ‘cure’ breakthrough. So, the search for a scalable cure continues.

### HIV Mice Cure Model

While most of the HIV eradication and cure research focused on humans, a recent study potentially eliminated HIV-1 in a subset of infected humanized mice (Dash et al., 2019; Gendelman and Khalili, 2019). In this study, human hematopoietic stem cells (HSC) reconstituted NOD.Cg-Prkdc^scid^ Il2rgt^m1Wjl^/SzJ (NSG) mice were used to produce human T cells that were vulnerable to HIV- 1 infection. The mice were engrafted with human CD34^+^ HSC isolated from cord blood, and HIV-1 was found to be highest (HIV RNA of 2.2 × 10^5^ copies/ml) in lymphoid compartments, with a notable decline in CD4^+^ T cells and an incline in CD8^+^ T cells. The main feature of this study was the utilization of a long-acting slow-effective release ART (LASER ART) which inhibited viral replication through hydrophobic lipophilic antiretroviral nanoparticles such as dolutegravir (DTG), rilpivirine (RPV), lamivudine (3TC), and abacavir (ABC). The HIV-1 subgenomic DNA fragments, spanning the long terminal repeats and the Gag gene was knocked out using adeno-associated virus serotype 9 (AAV_9_)-CRISPR/Cas9 system as a part of the HIV-1 elimination strategy.

The experiment was conducted using 33 NSG mice, of which four were sacrificed after 2 weeks of infection with 10^4^ TCID_50_ of HIV-1_NL__4__–__3_ to confirm HIV-1 infection. The remaining 29 infected mice were divided into four groups: (i) group 1 (*n* = 6) HIV-1 infected control and untreated, (ii) group 2 (*n* = 6) injected with AAV_9_-CRISPR/Cas9, (iii) group 3 (*n* = 10) received LASER ART, and (iv) group 4 (*n* = 7) administered with both LASER ART and AAV_9_-CRISPR/Cas9. At the end of the treatment procedure, viral rebound was observed in groups 2 and 3, which received a single treatment. Other than that, CD4^+^ T cells profile was reconditioned in groups that received LASER ART alone and LASER ART plus AAV_9_-CRISPR/Cas9 combination. Interestingly, viral rebound was absent in two (M4346 and M4349) out of the seven mice in group 4, whereas the remaining five mice showed detectable HIV viral load. Further DNA analysis using ultrasensitive semi-nested real-time qPCR, showed that the combination was more effective than single treatment. There was no detectable viral RNA in the two mice in either the plasma or tissue compartments; however, the predisposing factors that contributed to undetectable viral load among the two mice were not reported.

To validate the therapeutic HIV-1 elimination caused by LASER ART and CRISPR/Cas9, a similar experiment was conducted in 17 NSG mice, infected with 10^4^ TCID_50_ of HIV-1_ADA_. In this experiment, the mice were divided as follows: (i) group 1 (*n* = 4) HIV-1 infected and untreated, and (ii) group 2 (*n* = 13) administered with LASER ART, of which six mice received AAV_9_-CRISPR/Cas9 and seven mice did not receive CRISPR construct. Similar to the previous experiment, three mice treated with LASER ART and CRISPR/Cas9 showed no viral rebound, and there was an absence of HIV-1 RNA in two mice (M3319 and M3336). At the end of the study, splenocytes and bone marrow were isolated from HIV-1 mice with or without combination treatments for adoptive transfer. Notably, the mice which received the cells from the M3319 and M3336 showed absence of virus, providing a piece of evidence that LASER ART and CRISPR/Cas9 potentially eliminate HIV reservoir from tissue.

The usage of humanized mice model has been an essential contribution to the investigation of HIV biology, including life cycle, immunobiology, drug or vaccine target/delivery, tissue reservoir and persistency/latency (Policicchio et al., 2016; Marsden and Zack, 2017; Masse-Ranson et al., 2018). Even though the model is able to ‘mimic’ human cells present in peripheral blood or tissue, and exhibit both innate and adaptive response toward HIV infection, there are still some limitations (Nixon et al., 2017; Carrillo et al., 2018). The size and biology of the animal, which affects the blood volume for viral load, peripheral blood cells for functional assay, and the short lifespan are some of the issues to consider. Furthermore, the development of the GVHD after humanization with immune cells, limits the experimentation to only a few weeks. Despite these factors, this model still represents the natural targets of HIV infection and is being improved.

The potential therapeutic action of LASER and CRISPR/Cas9 was combined in the humanized mice as a strategy for HIV eradication and cure. The development of long-acting (LA) antiretrovirals (LA ARVs) provided an extended-release of the drug over weeks to months, and maintained sufficient drug concentration for antiviral activity (Benítez-Gutiérrez et al., 2018). Thus, LA ARVs addresses the limitations to ART, such as drug compliance, missed doses, drug resistance, gut toxicity and drug-drug interactions. Currently, two injectable nanosuspension LA ARVs are undergoing clinical development, including LA-rilpivirine (RPV) and LA-cabotegravir (CAB) (Gendelman et al., 2019). However, the LA ARVs can be rapidly metabolized and is not distributed extensively biologically. To counter such limitations, the LASER ART allows high drug loading capacities, extended circulation times, active targeting capabilities, enhanced drug dissolution rates and bioavailability, as well as reduced rapid plasma clearance (Edagwa et al., 2017). Most importantly, LASER ART potentially delivers the drugs to intracellular and tissue reservoir such as GALT, lymph nodes, genitourinary tract and the brain; while maintaining the therapeutic concentration over a long period of time.

On the other hand, the emergence of the CRISPR/Cas9 system to target HIV infection has also opened possibilities for HIV cure strategies (Zulfiqar et al., 2017). Several studies have used CRISPR/Cas9 to target host co-factors such as CCR5 and CXCR4, the viral DNA, as well as cleave the reverse-transcribed viral DNA (Das et al., 2019). The CRISPR/Cas9 introduces indels at the cleavage site, resulting in inactivation of virus and potentially contributing to HIV cure. However, in some instances, the mutations may cause a non-deleterious effect on HIV, prevent gRNA binding and result in viral escape. Thus, using a combinatorial CRISPR/Cas9 with dual gRNA can counter these effects. Currently, there are five CRISPR/Cas9 approaches that can be utilized against HIV infection (Saayman et al., 2015; Xiao et al., 2019): (i) targeting the pre-integrated proviral dsDNA directly, (ii) knocking out early-stage host co-receptor factor CCR5, (iii) cutting of the viral genome for LTR, (iv) cutting of viral genes responsible for release, and (v) targeting reactivation of latent cells, followed by subsequent ‘shock-and-kill’ strategies using ART and antiviral mechanisms.

**FIGURE 2:**
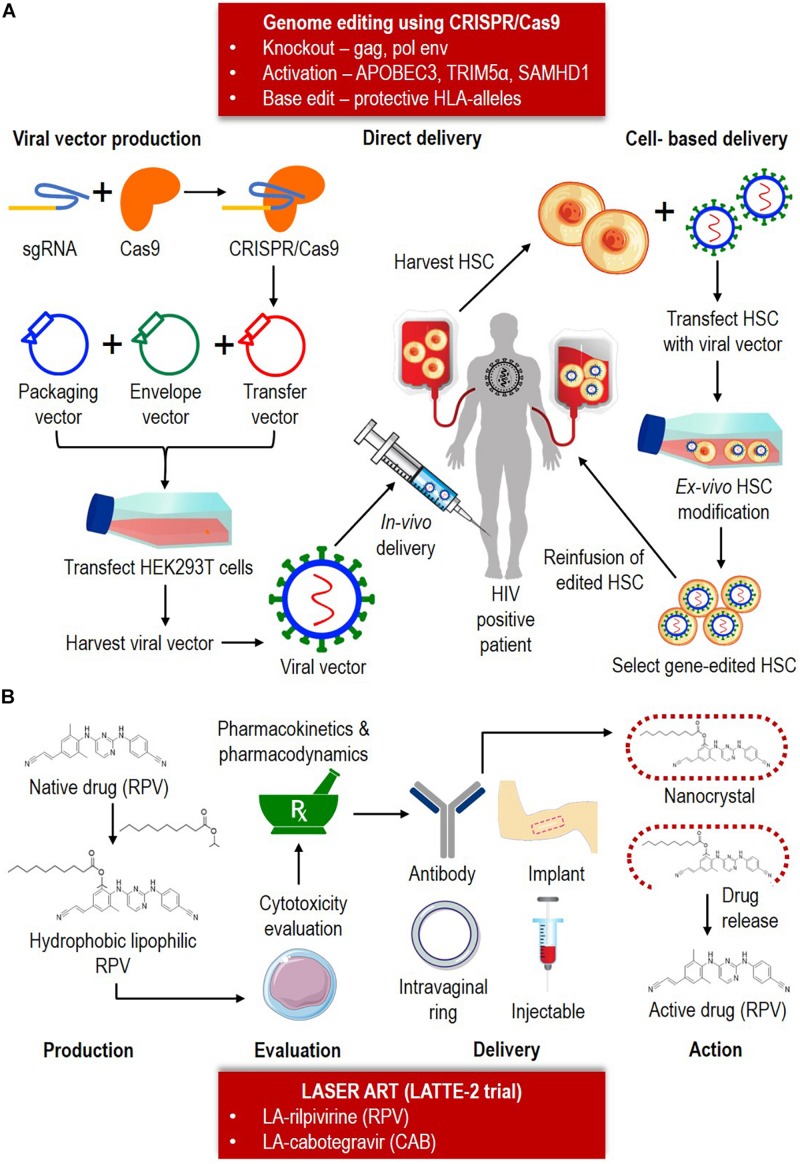
Combination of the CRISPR/Cas9 system and LASER ART as HIV eradication and cure strategies. **(A)** CRISPR/Cas9 can potentially be utilized for knockout, activation or base-editing depending on the nature of the therapy. CRISPR/Cas9 consists of a sgRNA and Cas9. CRISPR/Cas9 is typically delivered as a transgene in a transfer vector. The transfer plasmid, packaging plasmid and helper plasmid (envelope) is packaged to form a viral vector. The vector can be either delivered (ii) directly, by injecting *in vivo* into the infected patient, or (ii) cell-based, by first harvesting the hematopoietic stem cell (HSC) from the infected patient, and followed by transfecting the cells with viral vector containing the Cas9-sgRNA. After the *ex vivo* HSC modification, the clone containing the edited cells will be reinfused back into the HIV positive patient. **(B)** Based on LATTE-2 trial, currently two LASER ART are available (LA-RPV and LA-CAB). The production of LA-ARV involves conversion of native drug into hydrophobic lipophilic component. Cytotoxicity, pharmacokinetic and pharmacodynamic is performed to test the drug safety and efficacy. The drugs can be administrated by mean of antibody, intravaginal ring, implant or injection delivery. Upon reaching the target anatomical site, the nanocrystal will be broken down to release the active drug and further therapeutic action taken place.

Combination of LASER ART and CRISPR/Cas9 system as illustrated in Figure 2 may provide new strategies toward HIV eradication and cure in HIV positive patients. However, there are several few caveats to be considered in making LASER ART a success (Benítez-Gutiérrez et al., 2018; Gendelman et al., 2019): (i) the drugs should be produced with high intrinsic antiviral activity to reduce volume requirement, (ii) the drugs’ physio-chemical should allow for an extended-release at the site of action, (iii) injectable drug must able to prevent adverse effects (i.e., pain and discomfort), and (iv) in cases of treatment interruption (i.e., missed doses), a long-lasting subtherapeutic drug must be available to avoid drug resistance. For CRISPR/Cas9 system, the crucial factors for consideration are: (i) designing specific sgRNAs to reduce potential off-target effects, (ii) effective delivery of the large complex into infected cells, particularly across blood-brain barriers (i.e., nanoblades), (iii) inclusion of specific agents (i.e., latency-reversing agents) to eliminate HIV, and (iv) understanding the CRISPR/Cas9 mediated viral escape and developing effective strategies. Although the humanized mice model produced exciting results, it is important to understand why many of the experimental mice were not cured. More importantly, there are other aspects that are crucial for HIV elimination in humans, including the ability to deliver CRISPR construct into all parts of the human body.

## Hiv Cure Controversy: Lesson Learned

Bolstered by the success of curing HIV in animal models, a Chinese researcher, He Jiankui from the Southern University of Science and Technology, attempted to genetically engineer babies to be HIV-resistant, using CRISPR/Cas9 (Bi, 2018; Ryder, 2018). The edited babies, Lulu and Nana, were injected with CRISPR/Cas9 targeting the CCR5 co-receptor. In his YouTube video^1^, Jiankui claimed “protecting against HIV requires the simplest gene surgery, which is to remove just a few DNA letters,” and further added that “100 million people naturally have a genetic variation that disables CCR5. Jiankui induced non-homologous end joining (NHEJ) in the twins to knock out the function of CCR5 to make a 168 amino acid deletion in the loop region to prevent HIV binding. Even though Jiankui took some extra precautions to protect the babies, majority found it deeply disturbing and unethical (Cyranoski, 2018). He Jiankui presented his work at The Second International Summit on Human Genome Editing in Hong Kong (Davies, 2018) and answered questions posed to him^2^.

Before experimenting with the human embryos, he performed the preclinical studies on mice and monkeys, where the CRISPR-edited animals were born healthy. The preliminary studies also provided him with the knowledge of concentration, timing and number of injections to increase the editing efficiency in the embryo. Jiankui also performed the injection twice to reduce the likelihood of mosaicism, i.e., not all the cells in the early embryo was edited and resulted in a mosaic of different adult cells. Only after the verification of all these experiments did he move forward to prove his concept in human embryos. Lulu and Nana’s father was HIV positive, while their mother was negative. Upon intracytoplasmic sperm injection (ICSI), the CRISPR/Cas9 with the sgRNA was injected. The extent of editing was assessed using preimplantation genetic diagnosis (PGD) (Sanger sequencing and 30X whole genome sequencing), and the blastocysts were implanted into Grace, the mother. The implanted cells were monitored (MiSeq targeted sequencing, 609 cancer genes 40,000X ultra-deep sequencing) at the 12th, 19th and 24th weeks. He sequenced the placenta, umbilical cord and cord blood (Sanger sequencing, MiSeq targeted sequencing, 100X whole genome sequencing). Before the procedure, he also sequenced (Sanger sequencing, 30X whole genome sequencing) the parents of the twins, utilizing their genome as a reference sequence.

Jiankui demonstrated that (Ryder, 2018), Lulu only had one copy of mutation in her CCR5 gene (15 bp in-frame deletion), while Nana had mutations in both copies of her CCR5 gene (4 bp deletion, and 1 bp insertion). For Nana, the +1 mutation was similar to CCR5Δ32, but with an addition of 11 amino acids (underlined) (KSVSILEEFPDIKDSHLGAGPAAACHGHLLLGNPKNSASVSK), while the -4 mutation resulted in a shorter variant of the amino acid sequence (INSGRISRH). Both were predicted to produce non-functional truncated proteins. For the −15 mutation in Lulu, there was a five amino acid deletion near the HIV binding site (HFPYS) that was predicted to prevent HIV uptake. The truncated CCR5 in Nana could produce toxic and unwanted proteins as it was different from the CCR5Δ32 found in European populations (Bi, 2018). On the contrary, the CCR5 edited in Lulu would not provide protection. Apart from that, as the father was the one infected with HIV and HIV is rarely transmitted from father to baby vertically; thus, what Jiankui tried to prove seemed perplexing, as the babies could have been born HIV negative (Normile, 2018). However, Jiankui claimed that his reason for the editing was to protect the twins from probable infection later in life. He also stated that the goal of the project was to develop an AIDS vaccine. The experts condemned his action to be unethical and monstrous, as he put the babies at risk in later part of life, to achieve his goal.

Reviewing the timeline of Jiankui germline editing (Cyranoski, 2019), (i) June 2016, he initiated the project to edit genes in embryo, (ii) March 2017, couples were recruited for the experiment, whereby the father was an HIV carrier, (iii) November 2018, Lulu and Nana were born and there was also news on a pregnancy with a third gene-edited embryo, (iv) 25–26 November 2018, the MIT Technology Reviews and the Associated Press revealed the project to the public, (v) 28 November 2018, Jiankui’s work was scrutinized during the Second International Summit on Human Genome Editing, (vi) between November and December 2018, China’s National Health Commission began their investigation on Jiankui’s work, (vii) January 2019, the Guangdong’s Health Ministry disapproved of his conduct, and his university fired him, (viii) 18 March 2019, WHO established guidelines to govern and oversee human genome editing, and (ix) August 2019, the third gene-edited baby was expected, but the outcome was not made public.

He Jiankui and his team proposed a guide for the use of therapeutic *in vitro* fertilization gene surgery in human embryos (Jiankui et al., 2018) which were: (i) principle 1: mercy for families in need, early gene surgery was the only feasible way to save a child from lifetime suffering, (ii) principle 2: only for serious diseases, never for vanity, aesthetic, enhancement or gender selection, (iii) principle 3: respect a child’s autonomy, the rights of the child to live life freely and neither the parents nor the organization have obligations in any aspect, (iv) principle 4: genes do not define the purpose and the achievement; instead handwork, nutrition and support from society and loved ones are the determinants, and (v) principle 5: everyone deserves freedom from genetic disease, and the institution developing the cures have obligations to assist the families in need.

Although his core principles sounded noble, he did not adhere to some of them during his experiment, and the guide does not exempt Jiankui’s misconduct and ethical violation (Krimsky, 2019). Jiankui was found to violate the consensus released at the First International Summit on Human Gene Editing in 2015, that it would be “irresponsible to proceed with any clinical use of germline editing unless and until (i) the relevant safety and efficacy issues have been resolved, based on appropriate understanding and balancing of risks, potential benefits, and alternatives, and (ii) there is a broad societal consensus about the appropriateness of the proposed application.” He also failed to report his preclinical CRISPR work on mice and monkey, as well as the off-target effects sufficiently in the embryos. Furthermore, Jiankui did not obtain adequate information to reduce the risk of gene editing of embryos. He promoted his experiment as protection against HIV infection, which in reality, HIV can be prevented with other methods besides embryo gene editing. The attractive emolument offered to the parents (IVF payments, supportive care, and daily allowances totaling $40,000), could have blinded the judgment of the parents in weighing the risks and benefits. There were no initiatives to discuss the technical terminology to the parents or suggestions on the alternative methods of HIV prevention when obtaining consent. As an active board member in some companies in Guangdong and Beijing, there was also a conflict of interest for Jiankui, and it is uncertain if the experimentation done was for patient care or investment.

It is crucial to appreciate the lessons learned from Jiankui’s CRISPR babies (Bi, 2018; Cyranoski, 2019; Lovell-Badge, 2019): (i) ignorance to advice given by experts prior to initiating his experiments, and not taking seriously the risks associated with genome editing in germline (i.e., on-target alteration, off-target events, mosaicism), (ii) inadequate literature search of the consequences of editing CCR5 co-receptor (risk of CXCR4-tropic HIV-1 in Essen patient) including vulnerability of individuals to Western Nile and influenza viruses, and (iii) failure to obtain and adhere to the relevant regulation for ethical approval, as well as negligence toward science/clinical practices. Despite the guidelines on the “Human Genome Editing: Science, Ethics, and Governance” provided by the National Academies of Sciences, Engineering, and Medicine, it was not sufficient to stop Jiankui’s experiments and this calls for a moratorium of a fixed period of 5 years not to allow germline editing (Cohen, 2019b; Lander et al., 2019). Nevertheless, this case is an eye-opener on how things should be done and how all scientists have to follow the ethical workflow of using CRISPR/Cas9 for HIV eradication and cure research.

## Conclusion and Future Directions

Despite the evidence of the success of ART and newer approaches for HIV eradication and cure, there remains a few challenges and barriers (Lewin et al., 2011; Lederman et al., 2016; Elsheikh et al., 2019): (i) persistence of HIV in long-lived latently infected cells and anatomical reservoirs, (ii) persistence of infectious virus genome that does not effectively express virus protein and/or RNA (quiescence), (iii) the required amount of replacement and purging of host immune cells with HIV resistant cells (i.e., Berlin patient), and (iv) normalization of altered immune, inflammation and coagulation state of treated HIV infection. In short, HIV persistence, either pre-integration or post-integration latency, appears to be a significant challenge. However, the latter is more critical, as HIV DNA which integrates into the resting CD4^+^ T cells remain in the cells for long durations, inaccessible to ART or immune recognition.

There is however a small, important milestone achieved recently in HIV cure research pertaining to HIV persistence. Last year, an assay known as the intact proviral DNA (IPDA) enabled the estimation of intact proviral DNA with as few as 5 million CD4^+^ T cells, simultaneously detecting several conserved regions of the HIV genome such as intact packaging signal (PS) and the Rev-responsive element (RRE) within *env* (Bruner et al., 2019). Some researchers cautioned that this assay can only detect a small sub-genomic region (2% of the HIV genome), resulting in the risk of overestimation of intact proviral DNA (Falcinelli et al., 2019). Furthermore, the assay may also possibly quantify non-inducible provirus that may remain permanently latent for the lifetime of an HIV-infected patient. Therefore, while seemingly positive in overcoming the hurdle of efficient proviral quantification, the long-term benefit of this assay remains debatable for now.

There are a few existing successful and new interventions approaches that can be employed as a part of HIV eradication and cure (Passaes and Sáez-Cirión, 2014; Marsden and Zack, 2019): (i) bone marrow transplantation to create HIV resistant immune system, (ii) ‘kick-and-kill’ approach to remove long-lived latently infected cells, (iii) genome editing such as CRISPR/Cas9 to directly edit or excise integrated proviral DNA from the infected cells, and (iv) ‘block-and-lock’ approach to trap the latent proviral cells into a non-expressing state. The most effective therapy against the barriers to HIV cure, such as the latent reservoir, immune dysfunction, and viral persistence, will most likely only be tackled by using a combination therapy. In comparison to a monotherapy, the multi-step therapy could be composed of several approaches, including genetic inactivation of proviral genomes (CRISPR-Cas9), purging latent virus (the latency-reversing agents) and boosting anti-HIV immune response to ensure a thorough HIV eradication.

Although the target of HIV cure is clearer, it is not yet closer; as only the Berlin patient is free of virus, and HIV rebound occurred in Boston patients (Volberding, 2014) (refer Table 1). Reflecting this, the functional cure emerged as an alternative to the sterilizing cure (Durand et al., 2012). HIV eradication and cure research have also started focusing on unraveling the natural history of elite controllers (EC) (Tiemessen and Martinson, 2012). Originally, ECs was thought of as harboring defective viruses (Autran et al., 2011). However, it became clear that most of the ECs have high levels of circulating replication-competent virus, and host factors are the key to viral suppression (Cockerham and Hatano, 2015). Most of the cohort exhibit an overrepresentation of protective HLA class I alleles, providing a strong association that the immune system is vital in viral suppression (Baker et al., 2009). Among the HLA class I alleles, HLA-B had the most potent effect, specifically HLA-B^∗^57 and HLA-B^∗^27, which are associated with delayed disease progression. Interestingly, these HLA-B alleles have Bw4-motif family serospecificities, leading to targeted cell killing by natural killer (NK) cell receptor killer immunoglobulin-like receptor, three domains, long cytoplasmic tail, 1 (KIR3DL1). This mechanism enhances innate immunity alongside stimulation of adaptive immunity. CD4^+^ T cells secrete cytokines (IL-1 and IL-21) that potentially increase the release of cytotoxic molecules (granzymes, perforin, and granulysin) from CD8^+^ T cells, resulting in apoptosis of infected cells (Walker and Yu, 2013). Overall, ECs represent the best model for an HIV cure at the current stage, and it provides the alternative as to whether the cure should be disease-free or treatment-free state.

**TABLE 1:** Comparison among Berlin, London, Düsseldorf, Boston, Essen patients and Mississippi baby in terms of their HIV, malignancy and HSCT profiles.

**Parameters**	**Berlin**	**London**	**Düsseldorf**	**Boston A**	**Boston B**	**Essen**	**Mississippi**
Gender	Male	Male	Male	Male	Male	Male	Female
**HIV profiles**							
Age at HIV diagnosis	28-years-old	NA	NA	NA	NA	27-years-old	35-weeks-old
Baseline viral load	NA	180,000 copies/ml	29,400 copies/ml	NA	NA	NA	NA
Baseline CD4^+^ count	NA	290 cells/mm^3^	NA	NA	NA	NA	NA
ART regimen	EFV, FTC, TDF	EFV, FTC, TDF; EFV changed to RAL (during chemotherapy); RPV, 3TC, DTG (during HSCT)	FTC, TDF, DRV; DRV changed to RAL (before and during HSCT); ABC, 3TC, DTG (after HSCT)	EFV, FTC, TDF (before ART interruption); TDF, FTC, RAL, DRV/r (after ART interruption)	EFV, FTC, TDF (efavirenz-based combination), TDF, FTC, NFV, ABC (before HSCT); TDF, FTC, RAL (before and after HSCT/ART interruption)	LPV/r, TDF, FTC (before HSCT); LPV/r, 3TC, ABC and 3TC, ABC, RAL (after HSCT)	AZT (30 h of age); AZT, 3TC, NVP (31 h of age); AZT, 3TC, LPV/r (11 days of age)
Viral load after ART	Undetectable	1,500 copies/ml	Undetectable	NA	NA	Undetectable	19,812 copies/ml (31 h of age); 2617 copies/ml at (6 days of age); 516 copies/ml (11 days of age); 265 copies/ml (19 days of age); less than 48 copies/ml (29 days of age)
CD4^+^ count after ART	415 cells/mm^3^	NA	NA	NA	NA	NA	69% (8 days)
**Malignancy profiles**							
Age at malignancy diagnosis	40-years-old	NA	49-years-old	NA	NA	NA	
Types of malignancy	Acute myeloid leukemia	Hodgkin lymphoma	Acute myeloid leukemia	Hodgkin lymphoma	Diffuse large B-cell lymphoma	Anaplastic large-cell lymphoma	
Malignancy-related therapy	ATG, CSA, MMF, TBI, ARC, GO (first HSCT); CSA, MMF, TBI (second HSCT)	ABVD, BRESHAP, LEAM, LACE, MTX, CSA	ICE, HidAC, HAM, Flu, Treo, 5-AzaC, DLI	ABVD, ICE, GVD, Tac, Sir, MTX	R-CHOP, ABVD, ICE, Tac, Sir, MTX, DLI	ATG, CSA MTX	
**HSCT profiles**							
Viral load before HSCT	69,000,000 copies/ml (ART interruption)	Undetectable	Undetectable	144,000000 copies/ml	96,000000 copies/ml	Undetectable	
HSC donor	10/10 HLA match	9/10 HLA match	10/10 HLA match	7/8 HLA match	8/8 HLA match	10/10 HLA match	
CCR5Δ32 mutation	Yes	Yes	Yes	Yes	Yes	Yes	
GVHD	Skin (first HSCT)	Fever and GIT	NA	Skin, eye, liver and sclerodermatous	Skin, liver, oropharynx	Skin	
Chimerism after HSCT	At day 61 (first HSCT); at day 416 (second HSCT)	At day 30	∼At day 30 (first relapse); ∼At day 184 (second relapse)	At day 216	At day 220	NA	
Viral load after HSCT	Undetectable	Undetectable	Undetectable	Undetectable (at day 1266)	Undetectable (at day 652)	Undetectable	
HIV-1 antibody after HSCT	Gp120 and Gp41 positive, but declining; others negative	Gp160 slightly positive; others negative	Gp160 slightly positive; others negative	Decreased 5-fold	Decreased 10-fold	NA	
HIV-1 T cell after HSCT response	Loss of anti-HIV, virus specific, IFN- γ producing T cells	No response of T cells detected against Nef, Pol, Env	Strong CCR5-negative cells response against RT and Gag-P6	No PBMC activation	No PBMC activation	NA	
Tissue reservoir after HSCT	Undetectable (rectum)	NA	Undetectable (CSF, rectum, ileum, bone marrow, lymph node)	NA	Less than 2.4 copies/10^6^ cells (rectum)	Undetectable (rectum)	
ART interruption	One day before HSCT	On September 2017 (∼1 year after HSCT)	On November 2018 (∼4 years after HSCT)	4.3 years after HSCT	2.6 years after HSCT	7 days before HSCT	18 and 23 months of age
Viral load after ART interruption	Undetectable	Undetectable	Undetectable	4.2 million copies/ml	1.9 million copies/ml	93,390 copies/ml	Undetectable (21.9 months after interruption); 16,750 copies/ml (27.6 months after interruption)
Viral rebound	No	No	No	Yes	Yes	Yes	Yes
Viral remission	Undefined, declared as first person to be cured of HIV	18 months, premature to declare as second person to be cured of HIV	NA, premature to declare as third person to be cured of HIV	84 days	225 days	20 days	27 months
CXCR4-tropic HIV-1 rebound	No (CCR5 dependency)	Probably (positive *in vitro*)	NA	NA	NA	Yes	NA
Others	2X HSCT			New EFV-resistant mutation and acute retroviral syndrome	CSF had HIV-1 RNA levels of 269 copies/ml	Patient died with 7,582,496 copies/ml of HIV-1 RNA.	Positive gp160, gp120, gp41, p55, p24, p17, p66 during rebound

*Efavirenz (EFV); Emtricitabine (FTC); Tenofovir disoproxil fumarate (TDF); Raltegravir (RAL); Abacavir (ABC); Nelfinavir (NFV); Ritonavir-boosted darunavir (DRV/r); Zidovudine (AZT); Lamivudine (3TC); Nevirapine (NVP); Ritonavir-boosted lopinavir (LPV/r); Anti-thymocyte globulin (ATG); Cyclosporine-A (CSA); Mycophenolate mofetil (MMF); Methotrexate (MTX); Cytosine arabinoside (ARC); Gemtuzumab ozogamicin (GO); Adriamycin/oxorubicin, bleomycin, vinblastine, dacarbazine (ABVD); Brentuximab, etoposide, methylprednisolone, cytarabine, cisplatin (BRESHAP); Lomustine, etoposide, cytarabine. melphalan (LEAM); Lomustine, cytosine arabinoside, cyclophosphamide, etoposide (LACE); Ifosfamide, carboplatin, etoposide (ICE); High dose cytarabine (HidAC); High-dose cytosine arabinoside and mitoxantrone (HAM); Fludarabine (Flu); Treosulfan (Treo); Azacytidine (5-AzaC); Gemcitabine, vinorelbine, doxorubicin (GVD); Sirolimus (Sir); Tacrolimus (Tac); Cyclophosphamide, doxorubicin, vincristine, prednisone with rituximab (R-CHOP); Donor lymphocyte infusion (DLI); Total-body irradiation (TBI), High dose cytarabine (HidAC); Not available (NA).*

## Author Contributions

VK contributed to conception and wrote the manuscript. KT participated in drafting the review and revising it critically for important intellectual content. All authors reviewed the final manuscript submitted.

## Conflict of Interest

The authors declare that the research was conducted in the absence of any commercial or financial relationships that could be construed as a potential conflict of interest.

## Footnotes

1 https://www.youtube.com/watch?v=aezxaOn0efE

2 https://www.youtube.com/watch?v=tLZufCrjrN0
